# Desert Plant Endophyte Genome Database: a curated repository of endophytic bacterial genomes across arid ecosystems

**DOI:** 10.1093/database/baag020

**Published:** 2026-04-16

**Authors:** Sabiha Parween, Arun Prasanna Nagarajan, Amal K Alghamdi, Abdul Aziz Eida, Feras F Lafi, Luma Albanna, Nida Salem, Barakat Abu-Irmaileh, Zaid A Pirzada, Shahid Siddique, Ruben Garrido-Oter, Paul Schulze-Lefert, Maged M Saad, Heribert Hirt

**Affiliations:** Darwin 21 Desert Research Initiative, Biological and Environmental Science and Engineering Division (BESE), King Abdullah University of Sciences and Technology (KAUST), Building 2, Thuwal, Makkah Province, 23955-6900, Saudi Arabia; Darwin 21 Desert Research Initiative, Biological and Environmental Science and Engineering Division (BESE), King Abdullah University of Sciences and Technology (KAUST), Building 2, Thuwal, Makkah Province, 23955-6900, Saudi Arabia; Darwin 21 Desert Research Initiative, Biological and Environmental Science and Engineering Division (BESE), King Abdullah University of Sciences and Technology (KAUST), Building 2, Thuwal, Makkah Province, 23955-6900, Saudi Arabia; Department of Botany and Microbiology, College of Science, King Saud University, King Abdullah Road, PO Box 2455, Riyadh 11451, Saudi Arabia; Darwin 21 Desert Research Initiative, Biological and Environmental Science and Engineering Division (BESE), King Abdullah University of Sciences and Technology (KAUST), Building 2, Thuwal, Makkah Province, 23955-6900, Saudi Arabia; College of Natural and Health Sciences, Zayed University, Academic City Road, Dubai Academic City, Dubai, UAE; Department of Plant Protection, School of Agriculture, The University of Jordan, Queen Rania Al Abdullah Street, Al Jubeiha District, 11942, Amman, Jordan; Department of Plant Protection, School of Agriculture, The University of Jordan, Queen Rania Al Abdullah Street, Al Jubeiha District, 11942, Amman, Jordan; Department of Plant Protection, School of Agriculture, The University of Jordan, Queen Rania Al Abdullah Street, Al Jubeiha District, 11942, Amman, Jordan; Department of Microbiology, University of Karachi, University Road, 75270 Karachi, Pakistan; Department of Entomology and Nematology, University of California, One Shields Avenue, Davis, CA 95616, United States; Department of Plant-Microbe Interactions, Max Planck Institute for Plant Breeding Research, Carl-von-Linné-Weg 10, 50829 Cologne, Germany; Cluster of Excellence on Plant Sciences (CEPLAS), Heinrich-Heine-Universität Düsseldorf, Universitätsstraße 1, 40225 Düsseldorf, Germany; Earlham Institute, Norwich Research Park, Colney Lane, Norwich NR4 7UZ, United Kingdom; Department of Plant-Microbe Interactions, Max Planck Institute for Plant Breeding Research, Carl-von-Linné-Weg 10, 50829 Cologne, Germany; Darwin 21 Desert Research Initiative, Biological and Environmental Science and Engineering Division (BESE), King Abdullah University of Sciences and Technology (KAUST), Building 2, Thuwal, Makkah Province, 23955-6900, Saudi Arabia; Darwin 21 Desert Research Initiative, Biological and Environmental Science and Engineering Division (BESE), King Abdullah University of Sciences and Technology (KAUST), Building 2, Thuwal, Makkah Province, 23955-6900, Saudi Arabia; Max Perutz Laboratories, University of Vienna, Dr.-Bohr-Gasse 9, 1030 Vienna, Austria

## Abstract

Microbial communities associated with desert plants play a pivotal role in enhancing host survival under extreme environmental stressors, including drought, salinity, and nutrient limitation. The Desert Plant Endophyte Microbial Collection is one of the largest curated repositories of 2500 cultivable endophytic bacteria isolated from 23 native desert plant species across Saudi Arabia, Jordan, and Pakistan. Representing a broad spectrum of arid microhabitats from inland deserts and mountain wadis to coastal mangroves and date palm oases, the collection supports integrative studies on microbial ecology and plant–microbe interactions in water-limited ecosystems. A central component of this initiative is the Desert Plant Endophyte Genome Database, which currently hosts whole-genome sequences of 534 endophytic bacterial isolates annotated with extensive ecological metadata, assembly statistics, functional traits, and host associations. The database interface provides tools for genome exploration, metadata filtering, and functional gene mining, enabling users to identify taxa and traits of agronomic interest, particularly for applications in sustainable agriculture and sustainable desert revegetation. By combining genomic, ecological, and functional data, the Desert Plant Endophyte Genome Database serves as a foundational platform for the development of targeted microbial inoculants and fosters data-driven research into desert microbiomes and plant resilience mechanisms.

**Database URL:**  https://www.genomedatabase.org/.

## Introduction

Desert ecosystems represent some of the most challenging environments for plant life, characterized by intense heat, limited water availability, and high salinity. Despite these conditions, many native plant species have evolved physiological and biochemical strategies to persist and thrive. An emerging body of research has highlighted the critical role of plant-associated microbes, particularly endophytic bacteria, in mediating plant adaptation to such extreme abiotic stressors [1, 2]. These microbes, which reside within plant tissues without causing harm, contribute to host fitness by enhancing stress tolerance, facilitating nutrient acquisition, and synthesizing phytohormones and other growth-promoting compounds [3].

The Desert Plant Endophyte project was launched to systematically investigate and catalogue the endophytic microbial communities associated with native desert plants, with a specific focus on isolating strains with beneficial traits relevant to sustainable agriculture. By conducting extensive field sampling across arid and semi-arid habitats, including inland deserts, saline coastal zones, and mountainous wadis, the project has assembled one of the most comprehensive collections of cultivable endophytic bacteria from desert flora to date [4]. As a central outcome of this initiative, we introduce the Desert Plant Endophyte Genome Database, a curated and publicly accessible resource containing whole-genome sequences from over 500 bacterial strains. These strains were isolated from 23 ecologically diverse desert plant species, encompassing a broad taxonomic range that includes nitrogen-fixing legumes, halophytes, and pioneer grasses native to the country isolated from. The sequenced isolates span several major bacterial phyla, including Proteobacteria, Actinobacteria, Firmicutes, and Bacteroidetes, reflecting the taxonomic and ecological breadth of the Desert Plant Endophyte collection.

Each genome entry in the database is enriched with detailed contextual metadata, including plant host identity, geographic origin, isolation conditions, and functional annotations. Of particular interest are predicted plant growth-promoting traits (PGPTs) [5], such as genes associated with auxin biosynthesis, phosphate solubilization, and stress response. This enables users to perform genome mining for agricultural applications, comparative genomic analysis, and exploration of microbial adaptations to arid environments [6–11].

By integrating ecological metadata with high-quality genomic and functional annotations, the Desert Plant Endophyte Genome Database offers a resource platform for research in microbial ecology, host–microbe interactions, and climate-resilient crop development. We describe the structure, content, and navigation features of the database, and discuss its utility for researchers aiming to identify, compare, and deploy beneficial microbes for use in sustainable agriculture.

## Materials and methods

### Selection of plant hosts

The selection of plant hosts for the Desert Plant Endophyte endophytic bacterial collection was guided by a comprehensive review of ecological literature and targeted field surveys across arid and semi-arid landscapes. A total of 23 native plant species were chosen to represent a wide range of ecological functions and environmental adaptations. These species were selected for their prevalence in desert ecosystems, their resilience to abiotic stresses such as drought and salinity, and their ecological roles in soil stabilization, nutrient cycling, and vegetation recovery. The sampling sites encompassed diverse habitats, including inland deserts, saline coastal plains, and mountainous wadis across Saudi Arabia and adjacent regions.

To ensure broad phylogenetic coverage, the selected plant species were distributed across two major botanical classes, Magnoliopsida (17 species) and Liliopsida (6 species), spanning 10 botanical orders and 12 plant families. Key families included Fabaceae, Poaceae, Amaranthaceae, and Asteraceae, which are known to harbour diverse and functionally relevant microbial partners. The 23 species encompassed 19 genera, providing a diverse genetic and ecological foundation for microbial isolation (Supplementary Table 1).

The ecological characteristics of the host plants were used as a contextual framework for selecting species likely to harbour stress-adapted microbial communities. Leguminous plants such as *Acacia gerrardii, Indigofera argentea*, and *Astragalus tribuloides* were included due to their capacity for symbiotic nitrogen fixation [12, 13], which is essential in nutrient-depleted soils. Halophytic species like *Avicennia marina* [11], *Halothamnus bottae*, and *Zygophyllum simplex* were selected for their natural tolerance to salinity, offering insights into microbial adaptations in hypersaline environments. Pioneer species and soil-stabilizing grasses such as *Panicum turgidum, Dactyloctenium aristatum*, and *Erodium* spp. were chosen for their role in ecological succession and erosion control. In addition, the culturally and economically significant date palm, *Phoenix dactylifera* (Ajwa cultivar), was included for its long-standing association with desert agriculture and its potential as a model system for microbiome research [14, 15].

### Microbial isolation and identification

Root and shoot tissues were surface-sterilized and processed using standardized microbiological techniques as described on Alghamdi et al. [10, 11]. Culturable bacterial endophytes were isolated under sterile conditions, using a serial dilution technique. The serial dilutions were then plated onto different media for optimal bacterial isolation, including Luria–Bertani (LB), yeast extract (L7025, Sigma–Aldrich), Tryptic Soy Agar, and Zobell Marine agar. The purified bacteria were preserved in glycerol stocks for long-term storage. Strains were preliminarily characterized using phenotypic assays and 16S rRNA gene sequencing. A selected subset of strains underwent whole-genome sequencing to facilitate detailed functional and taxonomic analysis [4].

### Genomic sequencing, assembly, annotation, and phylogeny

Genomic DNA (gDNA) was extracted from pure bacterial cultures grown on LB agar plates incubated at 30 °C for 48 h. Total gDNA was isolated using the GenElute™ Bacterial Genomic DNA Kit (Sigma–Aldrich, Germany) according to the manufacturer’s instructions. DNA quality and concentration were assessed using spectrophotometry and agarose gel electrophoresis. Sequencing libraries were prepared using multiple platforms, including Illumina HiSeq, HiSeq 3000, MiSeq, PacBio RSII, and PacBio Sequel instruments. Illumina sequencing was performed using the Nextera XT DNA Library Prep Kit (or equivalent), producing 150 bp paired-end reads. PacBio libraries were constructed using the SMRTbell Template Prep Kit. Raw Illumina reads underwent quality control using fastp v0.20.1 [16], while PacBio reads were processed using Guppy or the SMRT Analysis Suite, with further filtering by Filtlong v0.2.1.

Genome assemblies were generated using different pipelines depending on sequencing technology: A4Pipeline (20 160 825) and SPAdes (v3.6–v3.15.5) [17] for Illumina reads, and HGAP (v4), smrtpipe v2.3.0.140936.p5, or HGAP.2 (v2.3.0) for PacBio data. Assembly quality metrics, including the number of contigs, N50 values, genome size, GC content, and completeness, were computed using QUAST v5.0.2 [18] and CheckM v1.2.2 [19]. The average genome completeness exceeded 95%, with contamination generally below 3%.

Genome assemblies were annotated using Prokka v1.14.6 using default bacterial settings. To construct a reliable species tree, core gene detection was performed across all taxa in the database in a single Roary v3.13.0 run [20] with default parameters, using all genomes together rather than separate species-specific runs. In Roary’s default definition, core genes are those present in 99% of the genomes in the dataset. The resulting core gene calls are not stored as a distinct feature layer in the database and are used only transiently to generate the species phylogenetic trees. Next, the core gene alignments were generated using MAFFT v7.525 [21], trimmed with trimAl v1.4 [22], and used to construct a maximum-likelihood phylogenetic tree with FastTree v2.xx [23] under the GTR + Gamma model. The tree was visualized in iTOL [24].

Functional annotation of assembled genomes was performed using Prokka v1.14.6 [25]. KEGG Orthology (KO) terms were assigned using eggNOG-mapper v2.1 [26], facilitating downstream functional analysis.

### Gene mining for potential plant growth-promoting traits

To functionally characterize plant-beneficial traits, we utilized the Plant-associated Bacteria (PLaBAse) resource [27], which curates over 6.9 million proteins representing 6900 unique PGPTs. Protein-level annotations were assigned using a combined BLASTp + HMMER approach against the PGPT database. Hits were filtered with a threshold of ≥40% identity and ≥70% query coverage to ensure confident trait assignments. This allowed systematic profiling of PGPTs across strains into Direct Effects, Indirect Effects, and Putative PGPTs. The PGPTs annotations were split into hierarchical level to reflect natural biological layering, from broad ecological roles down to gene-level functions.

### Database construction

To effectively store, manage, and deliver genomic and functional data, a MySQL relational database management system was employed. Custom database schema designs were implemented to ensure referential integrity across multiple tables encompassing genomic sequences, functional annotations, ecological metadata, and PGPT classifications. Schema normalization and indexing strategies were used to optimize query performance and facilitate scalable data retrieval [28]. A custom WordPress-based front-end was developed to allow seamless integration between the database and user-facing components. PHP scripts and templating logic dynamically rendered genome-specific records and facilitated secure downloads of associated files, including FASTA, GFF, and PGPTs annotations. The interface leveraged the Data Tables JavaScript library for building rich, client-side interactivity, including auto-complete search, column-specific filtering, pagination, and dynamic table rendering for fast browsing of large datasets [29]. The graphical user interface was built using standard web technologies: HTML5 for semantic content structure, CSS3 for responsive styling, and JavaScript for asynchronous interactions. These tools enabled a clean, responsive design that functions effectively across modern browsers and devices. For hosting, the platform is deployed on a Virtual Private Server with secure HTTPS access, ensuring high availability and rapid response times under concurrent user loads (Supplementary Fig. 1). Server-side caching and load-balancing mechanisms were employed to support smooth data access, even with increasing traffic volumes.

## Results and discussion

### Database structure and navigation

**Figure 1:**
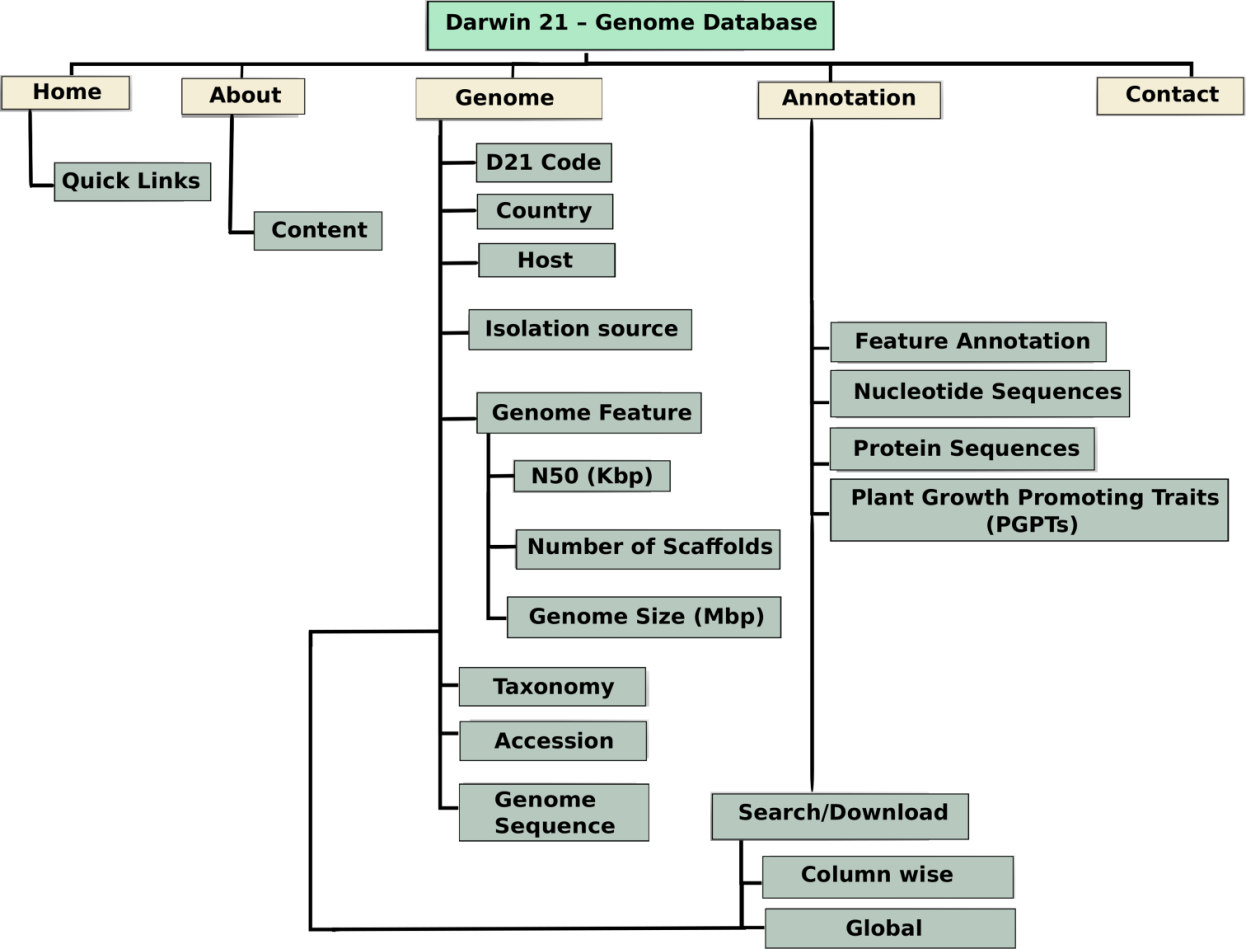
Overview of the Desert Plant Endophyte Genome Database architecture. The database is organized around genome-centric records, integrating assembly statistics, ecological metadata, functional annotations, and PGPT profiles. The main navigation includes a home page with summary views, a quick links panel for accessing downloads, statistics, and search functions and a separate annotation page. Users can interactively filter and sort data, perform global searches, and access downloadable annotation files. The interface supports dynamic exploration of taxonomy, gene content, and functional traits across the database.

The Desert Plant Endophyte Genome Database is organized as a genome-centric platform featuring multiple interactive sections and tools for exploring microbial data associated with desert plant endophytes, as shown in Fig. 1. The resource integrates comprehensive genome records that include detailed assembly metrics such as scaffold counts, N50 values, and genome size. Each entry links these data with extensive ecological metadata, including host plant information, country of isolation, sequencing platform, and accession numbers. Users can access and download a range of genome and annotation files for all strains, including protein sequences, and nucleotide sequences.

The database provides integrated feature annotations covering taxonomy, genome features, and PGPTs [27]. The interface emphasizes interactive tables where users can sort columns by attribute, filter records by values such as host plant, country, or Isolation source, and download genome data and associated PGPTs annotations directly. Contact and help options are always accessible from the main navigation panel. An intuitive navigation system supports column-wise filtering and global keyword searches and downloads across all records, enabling efficient retrieval and comparison of genome entries. Interactive tables allow users to sort and filter data dynamically and to move seamlessly between genome assembly details and functional annotations. Additionally, Supplementary Table 1 presents the taxonomic classification and ecological role of each plant host included in the study. Also, the whole-genome phylogenetic relationships among strains were inferred at the protein level from concatenated core gene alignments (Supplementary Fig. 2), providing high-resolution insights into strain relatedness, clonal clustering, and overall genomic diversity.

### Genome based access to potential plant beneficial traits

We systematically categorized microbial genes into distinct functional classes relevant to plant growth and stress resilience. Each microbial strain was profiled based on its encoded PGPTs summarizing three major trait categories: Direct Effects, Indirect Effects, and Putative PGPTs, and its related subcategories were organized hierarchically to reflect the multi-scale biological organization of microbial functions and to enable robust, confidence-aware functional inference.

Direct Effects encompass traits that directly enhance plant performance. These include genes involved in bio-fertilization processes such as nitrogen fixation, phosphate, and potassium solubilization, sulphur assimilation [30, 31], as well as carbon dioxide fixation [32]. Additionally, pathways contributing to phytohormone biosynthesis or modulation including auxins, gibberellins, cytokinins, and ethylene [33, 34] were identified. Additionally, microbial pathways involved in promoting plant development through signals such as root and shoot growth stimulation, branching, germination, and vitamin production were included. Indirect Effects include gene functions that enhance plant resilience through the mitigation of biotic and abiotic stressors. These involve biosynthesis of antimicrobial compounds, detoxification of heavy metals and xenobiotics, and activation of plant immune responses, including induced systemic resistance and systemic acquired resistance [35, 36]. The dataset also includes traits that facilitate microbial colonization of plant tissues, including chemotaxis, motility, surface attachment, and enzymatic degradation of plant-derived substrates [37, 38]. Putative PGPTs represent genes with the potential to play plant-beneficial roles that currently lack experimental validation. These were retained to ensure a broad functional inventory that supports hypothesis generation and further exploration. All annotated functional profiles for each strain are accessible for browsing and download through the Desert Plant Endophyte database.

### Desert Plant Endophyte - a valuable and pioneering genomic resource

Desert Plant Endophyte represents a unique and forward-looking genomic repository that integrates ecological context with high-resolution functional genomic data, with specific emphasis on microbial endophytes associated with desert plants. Unlike conventional databases, Desert Plant Endophyte interlinks high-quality genome assemblies with rich ecological metadata and experimentally relevant annotations, offering a comprehensive platform for investigating plant-microbe interactions in arid and extreme environments. Its trait-centric framework enables the identification of plant growth promoting genes across both experimentally validated and putative functional categories, providing unprecedented granularity for functional and ecological interpretation. The platform’s integration of interactive tools, customizable search queries, and user-friendly visualizations empowers researchers to derive biologically meaningful insights efficiently. As global interest in sustainable agriculture intensifies and climate-resilient crop systems continue to grow, Desert Plant Endophyte delivers critical infrastructure to support the exploration and application of microbial solutions aimed at enhancing plant resilience, nutrient acquisition, and environmental adaptation under abiotic stress conditions.

## Conclusions

In conclusion, Desert Plant Endophyte emerges as a critical platform for unlocking the genomic and functional potential of desert plant-associated microbiomes. By integrating high-quality genome data with ecologically meaningful annotations, it enables the systematic identification of plant-beneficial traits across diverse microbial taxa. Its interactive tools and trait-based framework not only facilitate in-depth exploration but also bridge the gap between basic microbial genomics and applied agricultural innovation. As the demand for climate-resilient and sustainable farming solutions grows, Desert Plant Endophyte will support future research and practical applications that harness the power of microbiomes to enhance crop performance under environmental stress.

## Supplementary Material

baag020_Supplemental_Files

## Data Availability

Most of the strains’ genomic data have also been submitted to NCBI under the projects PRJNA1042672, PRJNA1054060, PRJNA1076458, PRJNA312745, PRJNA313373, PRJNA313377, PRJNA345401, PRJNA352128, PRJNA398615, PRJNA708169, PRJNA740144, PRJNA827473, PRJNA929179, and PRJNA973967. The data underlying this article can be accessed at https://www.genomedatabase.org/.
