# Supplementary figures and images for: Desert Plant Endophyte Genome Database: a curated repository of endophytic bacterial genomes across arid ecosystems
Source: Database (Oxford). 2026 Apr 16;2026:baag020. doi: 10.1093/database/baag020 (PMC13092982; doi:10.1093/database/baag020)

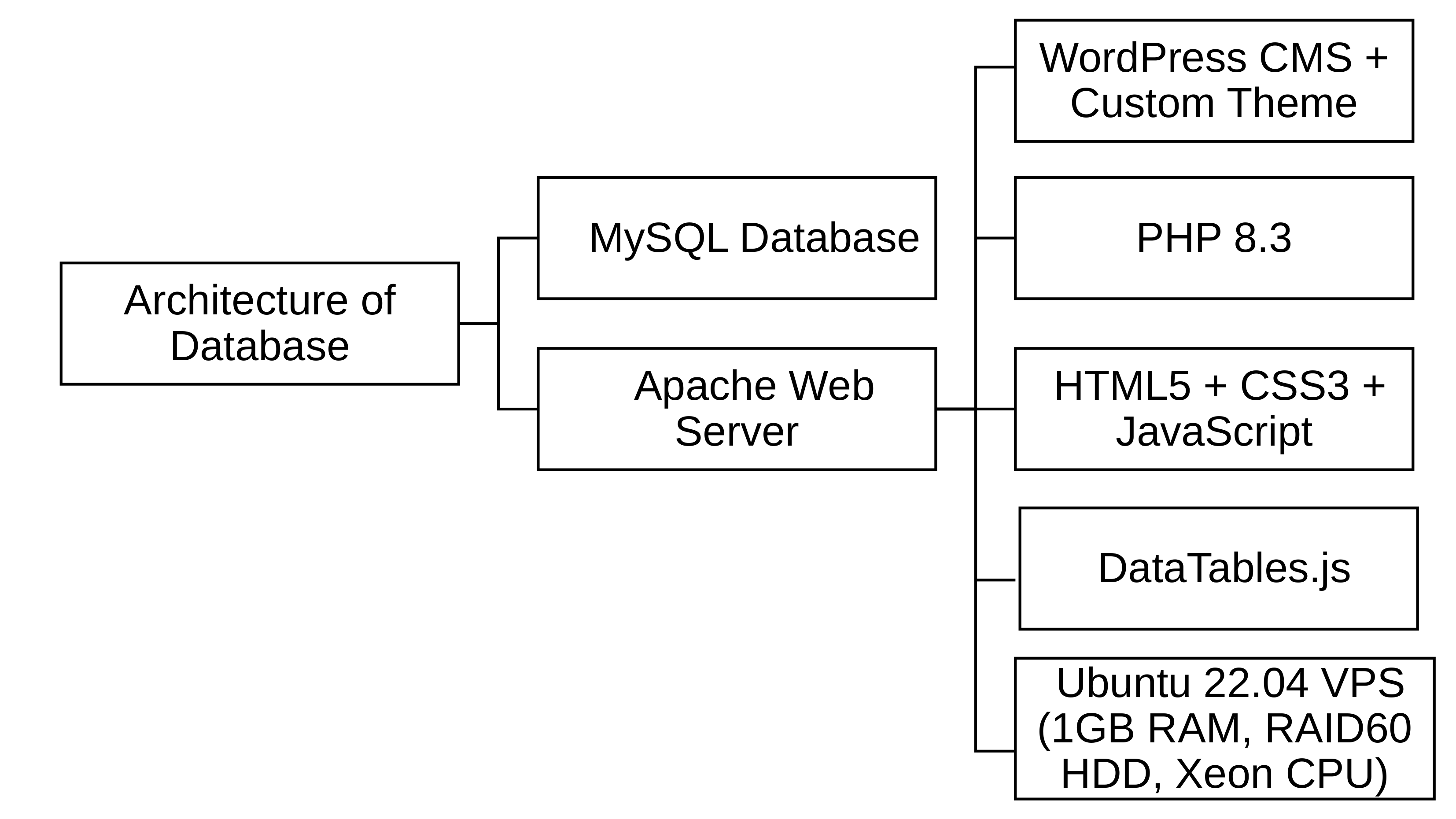

Supplement: baag020_Supplemental_Files [file baag020_supplemental_files.zip › FigS1.tif]

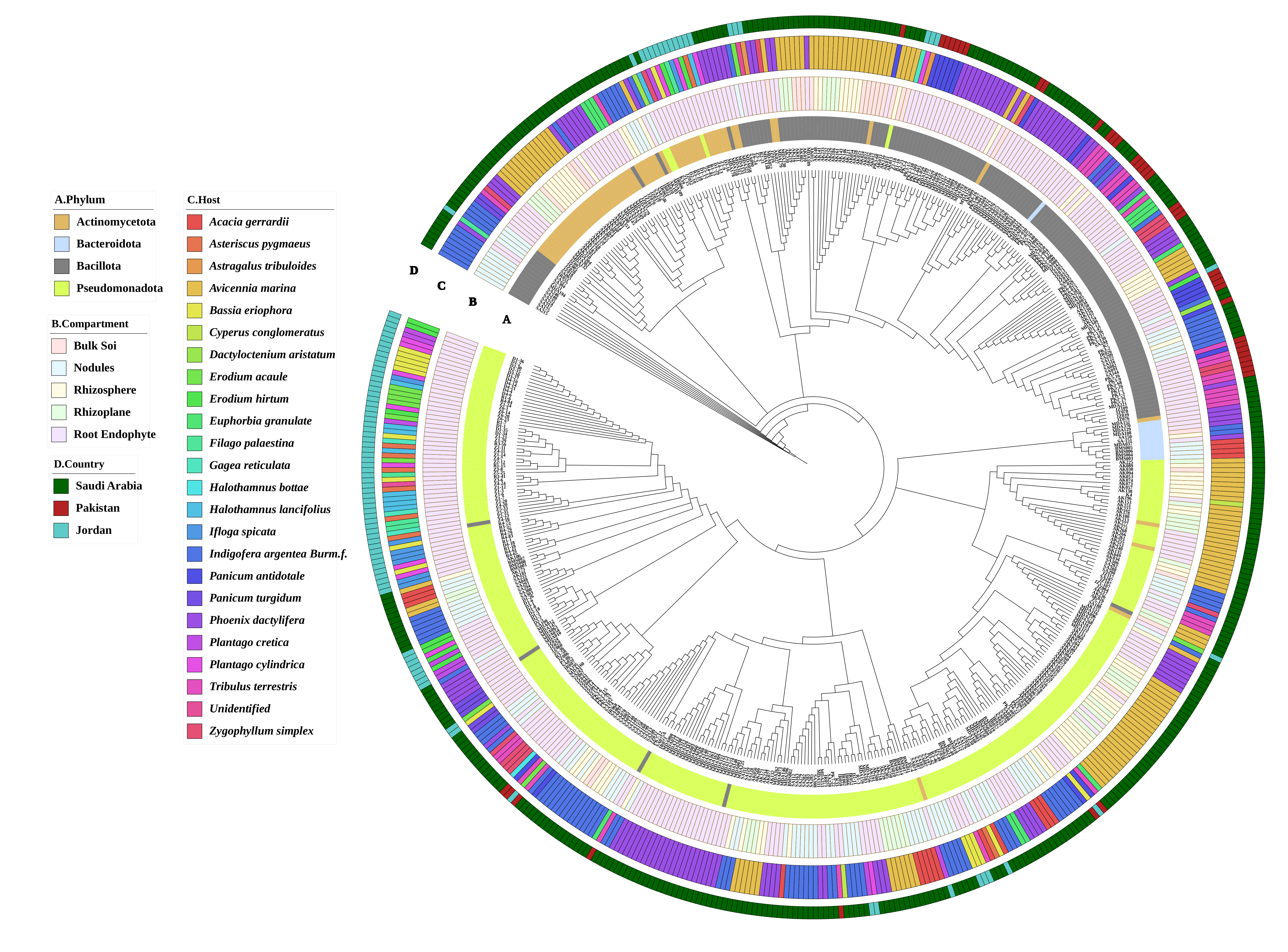

Supplement: baag020_Supplemental_Files [file baag020_supplemental_files.zip › FigS2.tif]
